# Understanding Waste Management Behavior Among University Students in China: Environmental Knowledge, Personal Norms, and the Theory of Planned Behavior

**DOI:** 10.3389/fpsyg.2021.771723

**Published:** 2022-01-12

**Authors:** Lingqiong Wu, Yan Zhu, Junqing Zhai

**Affiliations:** ^1^Research Center for Environment and Society, Hohai University, Nanjing, China; ^2^School of Economics and Management, Nantong University, Nantong, China; ^3^Shanghai Baoshan Center for Environmental Education, Shanghai, China; ^4^College of Education, Zhejiang University, Hangzhou, China

**Keywords:** theory of planned behavior, value-belief-norm theory, environmental knowledge, pro-environmental behavior, recycling

## Abstract

Previous studies have confirmed that individual waste management behavior is influenced by both rational-based and altruistic-oriented beliefs and attitudes. Scholars incorporated personal norms in Ajzen’s theory of planned behavior and confirmed its usefulness in predicting waste management behavior. However, limited attention has been paid to the interactions between the variables in the model. Scholars also commented that the cognitive dimension was largely neglected in the current socio-psychological framework of waste management behavior. This study intends to address this issue by incorporating environmental concern and environmental knowledge in the model and examining the psychological paths linking these variables to waste management behavior within the expanded model of planned behavior. Based on a cross-sectional survey among 434 university students in China, the results showed that subjective norms, perceived behavioral control, personal norms, and environmental knowledge were essential predictors of waste management behavior, whereas the direct effect of attitude was not statistically significant. Environmental concern and subjective norms could influence waste management behavior through personal norms. Environmental knowledge could influence waste management behavior indirectly through environmental concern, personal norms, and perceived behavioral control. Moreover, perceived behavioral control served as a mediator between the relationship of personal norms and waste management behavior.

## Introduction

Solid waste issue is one of the major issues in most countries at a global scale today (Abdel-Shafy and Mansour, 2018). It is estimated that global annual waste will reach 3.4 billion tons by 2050 (Kaza et al., 2018). If not collected or disposed appropriately, waste would pose significant threats to public health and the environment (Kaza et al., 2018). With an emerging consumer society and a large population of over 1.4 billion, China is among the countries facing the most serious effects of solid waste pollution (Zhou et al., 2017). To address this issue, China has implemented a series of laws and regulations on solid waste management, and initiated national programs to promote energy conservation awareness and environmentally responsible lifestyle in recent years. In 2017, the Chinese government issued the *Implementation Plan of the Household Waste Classification System*, which was regarded as a milestone for the institutionalization of public participation in recycling nationwide. One year later, a wider spectrum of waste management behavior which includes reduction, reuse, and recycling was highlighted in the trail version of *Citizen’s Ecological Environment Behavior Standard* (the Ministry of Ecology and Environment, 2018) to guide environmental education practice in China. If the most effective educational intervention is to be guaranteed, socio-psychological factors that are critical as well as the mechanisms through which these factors contribute to predicting waste management behavior should be studied.

In the field of environmental psychology, Ajzen (1991) theory of planned behavior (TPB) and Stern (2000) value-belief-norm model of environmentalism (VBN) represents two influential yet distinct approaches to understand pro-environmental behavior. The TPB explains pro-environmental behavior as a rational choice based on deliberate calculation of the expected costs and benefits of as well as the ability to perform the given behavior under certain social pressure. In contrast, the VBN understands pro-environmental behavior as a moral behavior determined by personal norms (i.e., internalized moral norms) with the latter being activated by environmental concern/beliefs and pro-social and/or environmental values. Nevertheless, pro-environmental behavior involves a complex decision-making process that is usually driven by multiple motives (Steg and Vlek, 2009; Onel and Mukherjee, 2017). Scholars have incorporated personal norms in the TPB and examined its role in predicting residents’ recycling in a variety of culture settings, such as in the United States (Onel and Mukherjee, 2017), in Australia (Chan and Bishop, 2013), and in China (Tang et al., 2011; Shen et al., 2020). The results from these studies consistently indicated that personal norms significantly predicted recycling intention or behavior over and beyond the TPB variables. However, the importance of personal norms in predicting recycling behavior in comparison with as well as the interactions between personal norms and the TPB variables has not yet been fully understood.

Recently, Morren and Grinstein (2021) examined the role of personal norms in the TPB in predicting pro-environmental behavior in different cultures using a meta-analytic structural equation modeling based on 255 samples. Their study suggests that rather than an antecedent of attitudes or a full mediator between subjective norms and intention, it would be more plausible to theorize personal norms as an antecedent of both intention and behavior; moreover, the relationship between personal norms and intention seems to be weaker in collectivistic than in individualistic cultures. Yet, previous research on waste management behavior in China mainly integrated personal norms as an antecedent of attitude in the TPB and focused largely on intention (Zhang et al., 2015; Xu et al., 2017; Shen et al., 2020). The direct effect of personal norms on waste management behavior in comparison with the TPB variables has rarely been addressed. To our best knowledge, only Tang et al. (2011) examined the importance of personal norms in the TPB model in predicting household recycling behavior in rural China. The population in their study was by large extent in pre-middle age (35–45 years) with a median level of education (i.e., 69% completed junior middle schools). Given the fact that young adults (aged 15–24 years) are identified as major targets for necessary interventions to foster a sustainable future (Fien et al., 2008), the present study intended to examine an expanded TPB model with personal norms as an antecedent variable of behavior in a specific young adult population (i.e., university students) in China. By focusing on this target population, the present study also attempted to provide a case with which the robustness of the relationship between personal norms and waste management behavior could be examined in well-educated young adult populations in China. Drawing on Stern (2000) VBN model, environmental concern was also included in the expanded model as an antecedent variable of personal norms.

**FIGURE 1:**
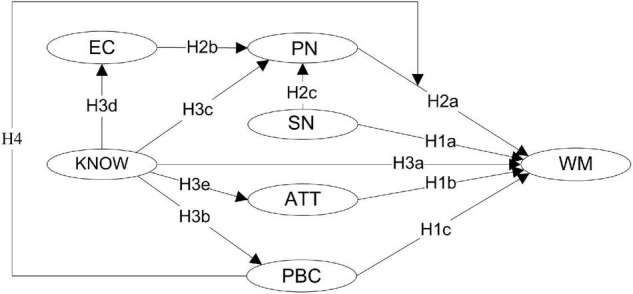
The expanded TPB model to be tested. WM, waste management behavior; SN, subjective norms; ATT, attitudes; PBC, perceived behavioral control; PN, personal norms; EC, environmental concern; KNOW, environmental knowledge.

Moreover, scholars have criticized that the role of environmental knowledge in shaping pro-environmental behavior was largely underestimated (Kaiser and Fuhrer, 2003; Steg and Vlek, 2009; Geiger et al., 2019). It is generally believed that although environmental knowledge *per se* is not a motive of pro-environmental behavior, it provides as essential cognitive basis upon which pro-environmental behavior can be developed (Stern, 2000; Schultz, 2002; Bamberg and Möser, 2007). Empirically, past research differentiated and identified two types of environmental knowledge that appeared to be critical in predicting pro-environmental behavior: environmental- and action-oriented knowledge. Environmental-oriented knowledge refers to an understanding of both ecological and social dimensions of the environment and environmental issues (also known as system or declarative knowledge, see Kaiser and Fuhrer, 2003 and Geiger et al., 2019), whereas action-oriented knowledge refers to knowledge of using action strategies to address environmental issues (i.e., knowledge of action strategies, see Hungerford and Volk, 1990) such as procedural and effectiveness/impact knowledge (Schultz, 2002; Kaiser and Fuhrer, 2003; Geiger et al., 2019). However, only very limited studies examined the role of environmental knowledge in shaping waste management behavior. More importantly, there is a paucity of research on the psychological path concerning how environmental knowledge contributes to shaping pro-environmental behavior. Recently, Geiger et al. (2019) examined the structure of environmental knowledge and found that environmental- and action-oriented environmental knowledge shared much in common and appeared to be a unidimensional factor associated tightly with the general knowledge of individuals. Thus, the present study intended to address this issue by integrating environmental knowledge (that comprises both environmental- and action-oriented knowledge) in the expanded TPB model and examining the indirect effect of this cognitive variable on waste management behavior through more specific attitudes in the expanded TPB model. The expanded TPB model to be tested is presented in Figure 1.

## Theoretical Framework

### Theory of Planned Behavior and Its Application in Waste Management Research

The TPB developed on the basis of Fishbein and Ajzen (1975) Theory of Reasoned Action (TRA). In the original model of TRA, intention plays the central role in determining a planned behavior; this variable is influenced by two critical factors including subjective norms and attitudes toward behavior. Subjective norms refer to perceived social pressure to perform the behavior in question, and attitudes toward behavior refer to personal evaluation of the behavior from a rational perspective (i.e., based on perceived benefits and costs the behavior may impose on individuals). Theoretically, the more intensive social pressure one perceives, and the more favorable consequence one expects a behavior can result in, the more intention one may have to perform the behavior, and hence, the more likely he or she would actually perform the behavior. Ajzen (1991) introduced the construct of perceived behavioral control (i.e., perceived ease or difficulty of performing the behavior) in the TPB as an antecedent variable of both intention and behavior. As this variable is based on an individual’s perception about both personal and environmental factors that would facilitate or hinder their ability to perform a behavior, the TPB is superior to the TRA in predicting behaviors in more specific and complicated contexts (Ajzen, 1991). Previous studies have applied the TPB and confirmed the usefulness of this model in predicting both intention to recycle (Taylor and Todd, 1995; Mannetti et al., 2004; Greaves et al., 2013; Botetzagias et al., 2015; Wan et al., 2017; Passafaro et al., 2019; Tian et al., 2019; Fedi et al., 2021) and recycling behavior (Tang et al., 2011; del Aguilar-Luzón et al., 2012; Onel and Mukherjee, 2017; Passafaro et al., 2019). Based on these studies, the following hypotheses are proposed:

H1a: Subjective norms positively predict waste management behavior.

H1b: Attitude positively predicts waste management behavior.

H1c: Perceived behavioral control positively predicts waste management behavior.

### Personal Norms and Environmental Concern

Personal norms refer to an individual’s feelings of moral obligation to perform a behavior (Schwartz, 1977). In contrast to the TPB, personal norms represent an altruistic perspective to human behaviors. As pro-environmental behaviors can be understood as altruistic behaviors with the purpose of either improving the well-being of living beings or conserving nature for its own sake or both, personal norms have been viewed as an essential factor in shaping pro-environmental behaviors (Van Liere and Dunlap, 1978; Stern, 2000). The relationship between personal norms and waste management behavior has been examined in different adult populations from a variety of cultures, for example in the United States (Thøgerson, 1996), in Brazil (Bertoldo and Castro, 2016), in Australia (Chan and Bishop, 2013), in European settings such as in Sweden (Hage et al., 2009; Andersson and von Borgstede, 2010), in Spain (del Aguilar-Luzón et al., 2012), and in Portugal (Bertoldo and Castro, 2016), and in Asian settings such as in China (Tang et al., 2011) and in Thailand (Janmaimool and Denpaiboon, 2016). It was found that personal norms significantly predicted waste management behavior in general (Janmaimool and Denpaiboon, 2016) or recycling behavior (Andersson and von Borgstede, 2010; Tang et al., 2011; Bertoldo and Castro, 2016) in specific even when the effects of the TPB variables were accounted for. Thus, the following hypothesis is proposed:

H2a: Personal norms positively predict waste management behavior.

Environmental concern (also known as the new ecological paradigm) is another critical factor influencing pro-environmental behavior from the altruistic perspective (Dunlap et al., 2000; Stern, 2000). This variable can be interpreted as a set of general beliefs about the environment and the relationship between humans and the environment (Dunlap et al., 2000). According to Stern (2000) value-belief-norm model of environmentalism, environmental concern provides a “folk” theory based upon the specific beliefs such as awareness of consequences (i.e., the perception of others’ welfare or needs) and ascription of responsibility (i.e., the apprehension of a sense of connection with others in need as an actor) are developed. As such, it contributes to the development of personal norms through awareness of consequences and ascription of responsibility. Previous studies have supported the sequential chain linking environmental concern to personal norms, and suggested environmental concern influences pro-environmental behaviors through personal norms (Kaiser et al., 2005; Onel and Mukherjee, 2017; Fornara et al., 2020; Zhang et al., 2020). Thus, the following hypothesis is proposed:

H2b: Environmental concern positively predicts personal norms.

Besides environmental concerns, social norms also provide an essential basis for the development of personal norms (Thøgerson, 1996; Bamberg and Möser, 2007). Social norms are common behavior standards that specify what is acceptable or appropriate within a society or reference group. They can be internalized and transmitted into personal norms through the process of socialization and social interaction (Schwartz, 1977). As subjective norms are felt social norms in a specific context held by a given reference group, it is reasonable to expect that subjective norms (including both injunctive and descriptive norms) would play a critical role in shaping personal norms. Some studies have examined the relationship between subjective norms and personal norms and found that subjective norms significantly predicted personal norms (Klöckner, 2013; Han et al., 2017; Fornara et al., 2020). Thus, the following hypothesis is proposed:

H2c: Subjective norms positively predicts personal norms.

### Environmental Knowledge

The importance of environmental knowledge has been emphasized widely in the field of environmental education (UNESCO-UNEP, 1977; Simmons, 1995; Hollweg et al., 2011). Hines et al. (1987) and Hungerford and Volk (1990) made initial efforts to incorporate environmental knowledge within a socio-psychological framework of pro-environmental behavior. Based on a meta-analysis of 128 studies on pro-environmental behavior, Hines et al. (1987) recognized knowledge of environmental issues and of action strategies as critical factors influencing pro-environmental behavior. The importance of these two specific kinds of environmental knowledge was highlighted once again in Hungerford and Volk (1990) model of citizenship behavior, as ownership, and empowerment variables in predicting pro-environmental behavior, respectively. Besides these two knowledge variables, they also integrated ecological knowledge as an entry-level variable of pro-environmental behavior. Both of their models were examined in a variety of adult populations both within and out of the United States (Sia et al., 1986; Sivek and Hungerford, 1990; Hsu and Roth, 1998; Marcinkowski, 1998; Cottrell, 2003). The results of these studies consistently showed that knowledge of action strategies or knowledge of environmental issues appeared to be a significant predictor of pro-environmental behavior, whereas the effect of ecological knowledge on pro-environmental behavior was relatively weak or insignificant. In the context of waste management, Janmaimool and Denpaiboon (2016) reported a significant effect of knowledge of action strategies on waste management behavior in a Thailand adult population. It was also found that environmental knowledge, either in general (Izagirre-Olaizola et al., 2015) or in association with environmental issues (Tang et al., 2011), significantly predicted recycling behavior even when the effects of the TPB variables were controlled for. Vining and Ebreo (1990) comparative study on recyclers with non-recyclers reported that recyclers were more knowledgeable about local recycling programs such as what items can be recycled and how to recycle these items (i.e., action-oriented knowledge). Based on these studies, the following hypotheses is proposed:

H3a: Environmental knowledge positively predicts waste management behavior.

In addition, it is theoretically plausible that environmental knowledge may also influence pro-environmental behavior indirectly through perceived behavioral control. In general, the status of having a comprehensive knowledge of environment science and actions based on past learning experience would lead to an increase in one’s confidence in performing pro-environmental behavior in a similar context (i.e., perceived behavioral control) in future. In particular, the more knowledgeable one perceives he or she is concerning how to make action strategies for issue-solving in a specific context (e.g., recycling), the more likely one may feel he or she has control over that action in that context. Thus, the following hypothesis is proposed:

H3b: Environmental knowledge positively predicts perceived behavioral control.

Environmental knowledge would also influence pro-environmental behavior indirectly through environmental concern. Since interrelatedness is a central concept of environmental knowledge (Enger and Smith, 2017), it is reasonable to expect that the more environmental knowledge one gains, the more likely he or she will understand environmental issues (including the causes and consequences) from a systematic perspective, and hence the more likely he or she would perceive nature and human-nature relationship from an ecological perspective. Two studies (Teksoz et al., 2012; Zhu, 2015) examined the relationships between environmental knowledge, environmental concern, and pro-environmental behavior, and found that environmental knowledge influenced pro-environmental behavior via environmental concern. Therefore, the following hypothesis is proposed:

H3c: Environmental knowledge positively predicts environmental concern.

Environmental knowledge (especially knowledge of environmental issues) would also foster a sense of responsibility for environmental improvement (Hungerford and Volk, 1990; Han et al., 2017), which would in turn contribute to the development of pro-environmental behavior. Empirically, Teksoz et al.’s (2012) study on environmental literacy found that environmental knowledge significantly predicted personal norms among university students. Bamberg and Möser (2007) meta-analysis also suggest that environmental knowledge would influence pro-environmental behavior through personal moral norms. Thus, the following hypothesis is proposed:

H3d: Environmental knowledge positively predicts personal norms.

Lastly, understanding of environmental issues in general and of waste issues in particular provides an essential cognitive basis upon which attitudes toward waste management behavior develop. For instance, the more knowledgeable one is about the impacts of waste pollution on environmental quality and human health, the more likely he or she would hold strong beliefs in the benefits of waste management behavior (e.g., reducing health risks because of waste pollution), and hence, the more favorable attitudes he or she would have toward waste management behavior. Empirically, a significant effect of environmental knowledge on attitudes was reported by Pivetti et al. (2020), who focused on recycling among an adult population in the Italian context. Therefore, the following hypothesis is proposed:

H3e: Environmental knowledge positively predicts attitudes.

### Perceived Behavioral Control and the Relationship Between Personal Norms and Waste Management Behavior

The relationship between personal norms and recycling can be influenced by contextual variables such as convenience. In light of the attitude-behavior-context (ABC) theory proposed by Guagnano et al. (1995), personal norms will play a critical role in determining recycling when only external barriers are at an intermediate level; in situations where recycling is too easy or too difficult, most people will recycle or not recycle no matter how weak or strong moral obligations they feel. Since perceived behavioral control captures a set of beliefs that “deal with the presence or absence of requisite resources and opportunities” (Ajzen, 1991, p. 196), it is reasonable to assume that this variable might influence the role of personal norms in predicting waste management behavior via *subjectively perceived* barriers. In particular, the more resources and opportunities one believes there are to support waste management behavior (i.e., less perceived barriers), the more likely personal norms would have a profound effect on waste management behavior, especially when external conditions make such behavior very difficult. Thus, the following hypothesis is proposed:

H4: Perceived behavioral control moderates the relationship between personal norms and waste management behavior.

## Materials and Methods

### Sampling

The present study took full-time undergraduate students in Jiangsu province in Eastern China as the case. Jiangsu represents one of the most developed provinces in China. By the end of 2019, Jiangsu had a resident population of 80.7 million, and reached a GDP scale of 9.96 trillion CNY (1.4 trillion USD), ranking first in GDP per capita and second in comprehensive competitiveness at the provincial level (Li et al., 2021). The sample was recruited from an online survey company (i.e., Wenjuanxin)^1^ via its survey system (Ma et al., 2019). The survey lasted for 1 week with a target of receiving at least 400 usable questionnaires. To encourage high response rate, participants were informed along with the consent letter at the beginning of the questionnaire that they had 25% chances to be rewarded with money of 5 CNY (approximately 0.775 USD) by lottery drawing after they completed the questionnaire. By the time the survey was terminated, 451 valid questionnaires were collected (71.2% of 625 questionnaires). Invalid questionnaires were excluded in light of the following criteria: questionnaires with (1) linearly/diagonally responses on all items throughout the entire set of the measures; (2) respondents beyond the undergraduate level (e.g., graduate students) or studied in a university/college outside of the target region; (3) questionnaires with either unknown or oversea IP addresses. At last, 434 usable questionnaires were obtained for data analysis. The final sample included 145 male (33.4%) and 289 female (66.6%) participants with ages ranging from 18 to 25 years (*M* = 20.50, *SD* = 1.28). The percentages of freshman, sophomores, juniors, and seniors were 44.7, 24.9, 20.3, and 10.1%, respectively. Among the participants, 53.9% had a major in science or engineering academic fields, whereas 46.1% had a major in social science or humanities academic fields. Basic characteristics of the final sample are shown in Table 1.

**TABLE 1:** Basic characteristics of the samples.

Characteristics	Frequency	Percentage
**Sex**		
Male	145	33.4
Female	289	66.6
**Age**		
Mean ± *SD*	20.50 ± 1.28 (18–25)	
**Grade level**		
Freshman	194	44.7
Sophomore	108	24.9
Junior	88	20.3
Senior	44	10.1
**Academic field**		
Science or engineering	234	53.9
Social science or humanities	200	46.1

### Survey Design

The questionnaire comprised seven individual scales measuring waste management behavior (WM) and the independent variables, which include subjective norms (SN), attitudes (ATT), perceived behavioral control (PBC), environmental concern (EC), personal norms (PN), and environmental knowledge (KNOW). Prior to the formal survey, a small-scale pilot survey was administered among two classes of undergraduate students (*N* = 56) who registered in a statistics and data analysis course taught by the first author. Feedback was collected to examine whether there were any problematic items with ambiguous meanings or incorrect expressions. Some minor modifications were done based on these feedbacks. The seven measures with item description are shown in Table 2.

**TABLE 2:** Item description on the measures.

Constructs	Items	Factor loading
Recycling behavior	REC1: Reuse recyclable materials in my daily life	0.793
	REC2: Classify recyclable waste, and then, properly dispose of them in the waste containers or sell them out in my daily life	0.813
	REC3: Reduce using/purchasing disposable products in my daily life	0.761
Subjective norms	SN1: Most people who are important to me think that I should reuse and/or recycle waste in daily life	0.858
	SN2: Most people who are important to me think that I should refuse to use or purchase disposable products in daily life	0.860
	SN3: Most people who are important to me are reusing and/or recycling waste in their daily life	0.813
	SN4: Most people who are important to me are taking steps to refuse to use disposable products in their daily life	0.843
Attitudes	ATT1: Reuse and/or recycle waste in daily life (*good* vs. *bad*)	0.653
	ATT2: Reduce using/purchasing disposable products in daily life (*good* vs. *bad*)	0.797
	ATT3: Reuse and/or recycle waste in daily life (*pleasant* vs. *unpleasant*)	0.821
	ATT4: Reduce using/purchasing disposable products in daily life (*pleasant* vs. *unpleasant*)	0.884
Perceived behavioral control	PBC1: Reuse and/or recycle waste in daily life (*have control* vs. *have no control*)	0.825
	PBC2: Reduce using/purchasing disposable products in daily life (*have control* vs. *have no control*)	0.879
Ecological worldview	EW1: The number of people living on earth is approaching the limit the earth can support	0.502
	EW2: When humans interfere with nature, it often produces disastrous consequences	0.731
	EW3: Humans are severely abusing the environment	0.594
	EW4: The balance of nature is very delicate and easily upset	0.617
	EW5: If things continue on their present course, we will soon experience severe ecological catastrophes	0.798
Personal norms	PN1: I feel I have personal obligation to reuse and/or recycle waste in my daily life for a better environment	0.920
	PN2: I feel I should take steps to prevent environmental problems by avoiding the usage of disposable products in my daily life	0.906
Environmental knowledge	KNOW1: Basic concepts of ecological system (such as energy flow and cycle of matter)	0.858
	KNOW2: Earth system science (such as ocean currents and earth climate)	0.868
	KNOW3: Natural resource and energy management (such as renewable and non-renewable resource)	0.894
	KNOW4: Environmental issues (such as marine pollution, air pollution, global warming, white pollution and related causes and consequences)	0.886
	KNOW5: Action strategies to address waste issues (such as ways of recycling/reuse and waste reduction)	0.851

Waste management behavior was measured using three items taping reuse, recycling, and reduce based on Swami et al. (2011). Participants were asked to evaluate how often they engaged in the three aspects of recycling in their daily life over the past year on a five-point Likert scale ranging from “never” to “always.” An earlier study showed that these three WM items together with green purchase behavior could be empirically differentiated from reducing energy use, conserving water, and choosing public transportation when going out, and could be regarded as high-cost pro-environmental behavior for university students in China (Wu and Zhu, 2021). Similarly, Wei et al. (2021) also differentiated waste reduction such as bringing reusable bags when shopping from electricity and water saving, and identified the former as costly saving behavior for Chinese university students.

Subjective norms, attitudes, and perceived behavioral control were assessed using self-developed items based on Ajzen (2002). Subjective norms were measures in terms of *injunctive* (i.e., the extent to which they believe that most people who are important to them think they should engage in waste management behavior) and *descriptive* (i.e., the extent to which they believe that most people who are important to them engage in waste management behavior) norms. For both types of norms, two items were developed and rated on a five-point Likert scale ranging from “strongly disagree” to “strongly agree.” For attitudes, participants were asked to assess their overall evaluation of waste management behavior in daily life on two five-point semantic differentials scales. These included (1) harmful/beneficial, which reflects the *instrumental* quality of recycling, and (2) unpleasant/pleasant, which pertains to the *experiential* quality of recycling. Perceived behavioral control was measured in a direct way using two items, which were rated on a five-point Likert scale ranging from “no control at all” to “complete control.”

Personal norms were measured using two items adapted from Thøgersen (2006). Environmental concern was measured using five items (i.e., NEP1, NEP3, NEP5, NEP13, and NEP15) from the revised Chinese version of NEP Scale (Wu and Zhu, 2021). These items pertain to balance of nature, limits-to-growth, and eco-crisis on the original NEP scale. Previous studies have demonstrated that these items were quite consistent and stable in representing an individual’s general belief of the environment and severity of eco-crisis across different populations in China (Wu and Zhu, 2021; Xu et al., 2021). The items were rated on a five-point Likert scale ranging from “strongly disagree” to “strongly agree.”

Environmental knowledge was measured in an indirect way (i.e., *perceived* knowledge) using items adapted from *the second author* (2015). Five items were used to assess the extent to which participants believe that they are knowledgeable about basic concepts of ecology, earth system science, natural resource and energy management, environmental issues, and action strategies in association with waste management. The items were rated on a five-point Likert scale ranging from “to little extent” to “to an extremely large extent.”

### Data Analysis

STATA 16.0 was used for basic descriptive analysis. The SmartPLS version 3.3.2 was used to test the hypotheses. Partial least squares structural equation modeling (PLS-SEM) was chosen because this approach makes no restrictive assumptions about the data distribution and has advantage to test more complex models with smaller sample sizes in comparison with covariance-based structural equation methods (CB-SEM) (Hair et al., 2019). In light of Hair et al. (2010) two-stage procedure for SEM analysis, the reliability and validity of the measurement model was examined in the first stage; then, the paths in the structural model were accessed in the second stage. Moderating effect was evaluated using a two-stage approach with mean-centered data. Significance of path coefficients in the models was examined using a bootstrap test with 5,000 subsamples.

## Results

### Measurement Model

Construct reliability and validity of the measurement model were firstly evaluated. As item NEP1 had a low loading (barely close to 0.5), the model was adjusted by removing this item. The results are shown in Table 3. Cronbach’s alphas and the values of composite reliability ranged from 0.627 to 0.921 and from 0.782 to 0.941, respectively, indicating acceptable construct reliability (i.e., above 0.6) (Hair et al., 2019). Convergent validity was examined using the average variance extracted (AVE). The AVE values for all but one construct (i.e., environmental concern) exceeded the threshold value of 0.5 (Hair et al., 2019); for the construct of environmental concern, the value (0.476) was quite close to 0.5, hence providing evidence for convergent validity.

**TABLE 3:** Reliability and validity of the measurement model.

Construct	α	CR	AVE	1	2	3	4	5	6	7
1. WM	0.698	0.832	0.623	**0.790**	0.600	0.363	0.537	0.259	0.504	0.464
2. SN	0.865	0.908	0.712	0.466	**0.844**	0.569	0.587	0.324	0.691	0.357
3. ATT	0.809	0.870	0.629	0.289	0.490	**0.793**	0.725	0.371	0.680	0.267
4. PBC	0.627	0.842	0.727	0.358	0.437	0.516	**0.852**	0.380	0.600	0.326
5. EC	0.645	0.782	0.476	0.188	0.264	0.284	0.269	**0.690**	0.460	0.230
6. PN	0.800	0.909	0.833	0.377	0.576	0.536	0.428	0.352	**0.913**	0.352
7. KNOW	0.921	0.941	0.760	0.378	0.323	0.234	0.251	0.195	0.304	**0.872**

Data indicating Fornell-Larcker criterion is shown below the diagonal; data indicating Heterotrait-monotrait (HTMT) is shown above the diagonal. CR, composite reliability; AVE, average variance extracted. The values of square root of AVE are shown in the diagonal in bold. WM, waste management behavior; SN, subjective norms; ATT, attitudes; PBC, perceived behavioral control; PN, personal norms; EC, environmental concern; KNOW, environmental knowledge.

The discriminant validity was assessed using Fornell-Larcker and heterotrait-monotrait (HTMT) estimates. For a construct with good discriminant validity, the value of the square root of AVE for this construct should be greater than the correlations of this construct with any other constructs and the value of the HTMT ratio should be smaller than 0.85 (Hair et al., 2019). As shown in Table 3, all constructs had acceptable discriminant validity, with the values of the square root of AVE for any given construct were greater than the corresponding correlations in question, and the values of the HTMT ratio ranging from 0.230 to 0.725. Moreover, the variance inflation factors (VIF) of all constructs (i.e., inner VIF values) ranged from 1.000 to 1.832 (lower than the recommended value of 5), suggesting that multicollinearity was not a severe issue in this study.

### Structural Model

The primary purpose of this study was to examine the role of personal norms in predicting waste management behavior within the expanded TPB model among the young adult population in China. The importance of environmental knowledge in shaping waste management behavior as well as the psychological path that links environmental knowledge to waste management behavior within the expanded TPB model was also explored. For comparative purposes, three models were established. The original model included the three TPB variables only; the second model included personal norms as an antecedent variable of waste management behavior based on the first model; the third model (i.e., the complete model) integrated environmental knowledge as an antecedent variable of waste management behavior, perceived behavioral control, environmental concern, personal norms, and attitudes based on the second model. The complete model explained 31.8% of the variance in waste management behavior, indicating weak-to-moderate explanatory power (Hair et al., 2019).

Basic measures of model fit in PLS-SEM include the standardized root mean square residual (SRMR), the unweighted least squares discrepancy (*d*_*ULS*_), the geodesic discrepancy (*d*_*G*_), and the normed fit index (NFI). The values of SRMR, *d*_*ULS*_, *d*_*G*_, and NFI for the estimated and the saturated models were 0.139 and 0.066, 5.836 and 1.301, 0.655 and 0.520, and 0.684 and 0.726, respectively. Although the SRMR for the estimated model exceeded 0.08 (a threshold indicating a good fit for CB-SEM) and the values of NFI for both estimated and saturated models were smaller than 0.9 (a threshold indicating acceptable fit for CB-SEM), Hair et al. (2019) suggest these guidelines should be regarded as very tentative for PLS-SEM. The reason lies in that the PLS-SEM algorithm is not based on minimizing discrepancy between observed and estimated covariance matrices as it does in CB-SEM; rather, the primary aim of model estimation in PLS-SEM is to maximize the explained variance of endogenous constructs (Hair et al., 2019). Hence, a global fit measure (*GoF*) for PLS-SEM, which is defined as the geometric mean of average AVE and average *R*^2^ for endogenous constructs (Wetzels et al., 2009), was used as supplementary evidence of model fit in the present study. The *GoF* value was 0.34 for the complete model, which is close to the cut-off value of 0.36 for large effect size of *R*^2^ (the cut-off value for medium effect size is 0.25), suggesting acceptable model fit of the complete model (Wetzels et al., 2009). The direct and indirect effects of the variables in the complete model are shown in Table 4.

**TABLE 4:** Standardized path coefficients of direct and indirect effects in the structural model.

Paths	β	*t*	*p*	Bias-corrected 95%CI^a^		Hypothesis Check
				LB	UB	
***Direct effects***						
WM ← SN	0.276***	4.656	0.000	0.164	0.392	H1a: Supported
WM ← ATT	−0.035	0.641	0.521	−0.139	0.073	H1b: Not supported
WM ← PBC	0.172**	2.832	0.005	0.051	0.287	H1c: Supported
WM ← PN	0.132*	2.374	0.018	0.025	0.243	H2a: Supported
PN ← EC	0.202***	4.778	0.000	0.116	0.282	H2b: Supported
PN ← SN	0.487***	13.009	0.000	0.411	0.555	H2c: Supported
WM ← KNOW	0.216***	4.539	0.000	0.123	0.306	H3a: Supported
PBC ← KNOW	0.253***	5.501	0.000	0.154	0.337	H3b: Supported
PN ← KNOW	0.108**	2.580	0.010	0.028	0.190	H3c: Supported
EC ← KNOW	0.195***	4.260	0.000	0.098	0.278	H3d: Supported
ATT ← KNOW	0.244***	5.609	0.000	0.157	0.326	H3e: Supported
WM ← PN × PBC	0.106**	3.030	0.003	0.033	0.169	H4: Supported
***Indirect effects***						
WM ← PN ← SN	0.065*	2.350	0.019	0.013	0.121	
WM ← PN ← EC	0.027*	2.061	0.039	0.006	0.057	
WM ← PBC ← KNOW	0.043*	2.324	0.020	0.012	0.084	
WM ← PN ← KNOW	0.014^+^	1.653	0.098	0.002	0.038	
WM ← PN ← EC ← KNOW	0.005^+^	1.779	0.075	0.001	0.013	

^+^p < 0.1; *p < 0.05; **p < 0.01; ***p < 0.001. ^a^Bias-corrected 95%CIs were calculated using a bootstrap test with 5000 subsamples. WM, waste management behavior; SN, subjective norms; ATT, attitudes; PBC, perceived behavioral control; PN, personal norms; EC, environmental concern; KNOW, environmental knowledge.

#### Three Theory of Planned Behavior Variables as Predictors of Waste Management Behavior

**FIGURE 2:**
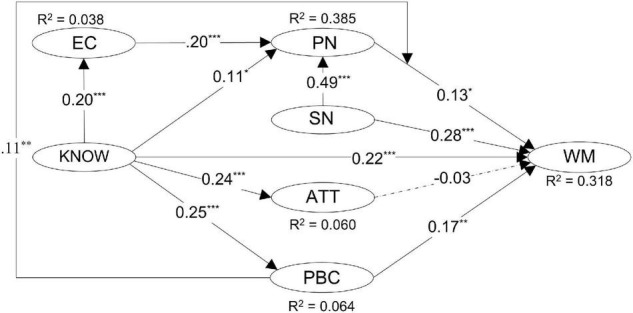
Standardized estimates for the paths in SEM model. Insignificant effects are illustrated in dashed line. *p < 0.05; **p < 0.01; ***p < 0.001. WM, waste management behavior; SN, subjective norms; ATT, attitudes; PBC, perceived behavioral control; PN, personal norms; EC, environmental concern; KNOW, environmental knowledge.

To begin with, attitude had no significant effect on waste management behavior when only the three TPB variables were included in the model (see Figure 2). The effects of subjective norms (β = 0.28, *p* = 0.000) and perceived behavioral control (β = 0.17, *p* = 0.000) remained significant after personal norms and environmental knowledge were added to the model. The results of the bootstrap test also indicated significant effects of these two TPB variables on waste management behavior. Hence, hypotheses H1a and H1c were accepted, but H1b was rejected.

#### Personal Norms as Predictor of Waste Management Behavior

Personal norms positively and significantly predicted waste management behavior (β = 0.16, *p* = 0.004). Adding this variable in the original model could increase the explanatory power from 24.8 to 27.8%. The bootstrap test showed that its effect remained significant (β = 0.13, *p* = 0.018) after environmental knowledge was added into the model. Thus, hypothesis H2a was supported. This research also examined the relationships of personal norms with environmental concern and subjective norms. The results show that both environmental concern (β = 0.20, *p* = 0.000) and subjective norms (β = 0.49, *p* = 0.000) positively and significantly influenced personal norms in the complete model. The bootstrap test showed the effects of these two variables on personal norms were significant. So, hypotheses H2b and H2c were supported.

#### Environmental Knowledge as Predictor of Waste Management Behavior

As expected, environmental knowledge positively and significantly predicted waste management behavior (β = 0.22, *p* = 0.000). The bootstrap test showed its effect was significant. Thus, hypothesis H3a was accepted. In addition, this variable also significantly influenced perceived behavioral control (β = 0.25, *p* = 0.000), environmental concern (β = 0.20, *p* = 0.000), personal norms (β = 0.11, *p* = 0.010), and attitudes (β = 0.24, *p* = 0.000). The results of the bootstrap test showed similar results. Thus, hypotheses H3b, H3c, H3d, and H3e were supported. Moreover, the bootstrap test also indicated that the indirect effect for the path from environmental knowledge through perceived behavioral control to waste management behavior (i.e., KNOW → PBC → WM) was significant (Table 4). The indirect effects for the paths of KNOW → PN → WM and KNOW → EC → PN → WM appeared to be significant though very weak at a significant level of 0.1, too. The three paths jointly accounted for 20% of the total effect of personal norms on waste management behavior.

#### Perceived Behavioral Control as a Moderator

Finally, this study examined the role of perceived behavioral control as a moderator influencing the relationship between personal norms and waste management behavior. It was found that the interaction term of PN × PBC had a positive and significant effect on waste management behavior (β = 0.11, *p* = 0.003). In particular, the result of the slope analysis illustrates that personal norms are important to individuals who have a relatively high level of perceived behavioral control. The significance of the effect was confirmed by the result of the bootstrap test. Thus, hypothesis H4 was supported.

## Discussion

This study integrated personal norms, environmental concern, and environmental knowledge in the TPB model and applied this model to understand waste management behavior among university students in China. The results showed that subjective norms, perceived behavioral control, personal norms, and environmental knowledge significantly predicted waste management behavior. Attitudes had no significant effect on waste management behavior. Environmental concern influenced waste management behavior through personal norms. The effect of subjective norms on waste management behavior were partially mediated by personal norms. Environmental knowledge could also influence waste management behavior indirectly through environmental concern, personal norms, and perceived behavioral control. Moreover, perceived behavioral control moderated the relationship between personal norms and waste management behavior. The results suggested that waste management behavior of university students in China could be viewed as context-dependent and morally driven practice. The results also added new evidence to support the importance of personal norms and environmental knowledge in shaping waste management behavior.

To begin with, it is interesting that among the three TPB variables, attitudes had no significant effect on waste management behavior, even when only the three TPB variables were taken into consideration. This finding differs from that in Tang et al. (2011), in which a significant effect of attitude was reported on household recycling behavior in rural China, but was consistent with Wan et al. (2017), who applied the TPB in understanding the public’s intention of recycling in Hong Kong, China. In comparison with Tang et al. (2011), the insignificant effect of attitudes on waste management behavior in both the present and Wan et al. (2017) studies might be partly attributed to a ceiling effect in measuring attitudes given the specific institutional contexts of waste management in these studies. Unlike Tang et al. (2011), who reported recycling had not yet been put forth by the local government upon the time their study was carried out, waste reduction and recycling had been formally emphasized and subsidized for both the present and Wan et al. (2017) studies. This means that the samples in these two studies were situated in a much more favorable context for waste reduction and recycling, hence they would have more positive attitudes toward such practices. Actually, the samples in the present study reported consistently high scores (i.e., 4.24–4.65) across all of the four attitude items, indicating that they, in general, regarded waste management behavior as a pleasant practice and believed that the participation in waste management could bring personal benefits. There is no direct evidence to show the sample in Wan et al. (2017) work had uniformly high levels of attitudes toward recycling. However, an earlier study on recycling (Chan, 1998) as well as a recent study on environmental concern in Hong Kong (Cheung et al., 2015) suggest that the residents in this area would have positive attitudes toward recycling. Jointly, the inconsistent results in association with the relationship between attitudes and waste management may suggest that the role of attitude on pro-environmental behavior might be vulnerable to the specific context or population involved in different studies.

It should also be noted that although the effect of attitude in Tang et al. (2011) study was significant, its effect was weaker than those of subjective norm and self-efficacy. Hence, the finding concerning the superior role of subjective norms in predicting waste management behavior in comparison with attitudes in the present study collaborates those in previous studies in supporting a cultural explanation of social behaviors. That is, subjective norms play a more important role than attitudes do in personal decision-making in collectivistic cultures than in individualistic cultures (Tang et al., 2011; Morren and Grinstein, 2021). It would be especially the case given the fact that the majority of university students in mainland China live a dormitory life on campus. As both subjective norms and perceived behavioral control are subjective to external circumstances in a more direct way than attitudes, the findings imply that waste management behavior would be understood as context-dependent pro-environmental practice for university students.

Secondly, personal norms represent an altruistic perspective to understand pro-environmental behavior. Previous studies have added this variable in the TPB in predicting both intention to recycle (Botetzagias et al., 2015; Wan et al., 2017) and recycling behavior (Tang et al., 2011; Onel and Mukherjee, 2017). In accordance with the findings of these studies, a significant effect of personal norms was found on waste management behavior of university students in this study. This suggests that moral concern would be an essential factor driving waste management behavior in general. Moreover, the finding concerning the effect of personal norms on waste management behavior in comparison with those of subjective norms and perceived behavioral control is similar to that reported by Tang et al. (2011), who took the adult population in rural China as the case. Drawing on these findings, it could be inferred that the role of personal norms in predicting waste management behavior within the TPB may be robust to individual differences in age and educational level in the Chinese context.

In addition, the present study found that subjective norms, environmental concern, and environmental knowledge significantly predicted personal norms. This means that if people perceive more intensive social pressure to waste management, hold stronger beliefs in limits of growth and eco-crises, and are more knowledgeable of both environment issues and action strategies to perform waste management practices, they would be more likely to develop a sense of moral obligation to engage in waste management behavior. Comparatively, subjective norms had the strongest effect on personal norms among the three variables. As subjective norms capture expectations from important others such as parents, teachers/tutors, peers who usually play important roles in socialization, informal environmental virtue/moral education during the course of socialization process would have profound influence on the development of pro-environmental norms among Chinese university students. Further studies are needed to examine the relationships of these variables with personal norms in a wider range of university student populations as well as other adult populations in mainland China.

The present study also contributed to the body of environmental literacy literature by probing the role of environmental knowledge in predicting waste management behavior within the expanded TPB model. Consistent with previous studies on environmental knowledge (Seacat and Northrup, 2010; Tang et al., 2011; Izagirre-Olaizola et al., 2015), the present study found that environmental knowledge significantly predicted waste management behavior over and beyond the TPB variables and personal norms. Moreover, the findings revealed that besides direct effect, environmental knowledge also influenced waste management behavior indirectly through the paths of KNOW → EC → PN → WM, KNOW → PN → WM, and KNOW → PBC → WM. Thus, we argue for a theoretical position of environment knowledge as critical antecedents of attitudinal variables within a social-psychological framework of waste management behavior. Noting that the indirect effect accounted only for 20% of the total effect, it is also strongly recommended that future studies examine other psychological mechanisms linking environmental knowledge and waste management behavior.

More importantly, the present study made a novel contribution to test the moderating effect of perceived behavioral control on the relationship between personal norms and waste management behavior. As expected, the results revealed that perceived behavioral control positively moderated the effect of personal norms on waste management. This means that in an inconvenience context in which personal norms play a critical role in driving waste management (Guagnano et al., 1995; Hage et al., 2009; Moore and Boldero, 2017), the stronger one believes he or she has the ability to overcome external barriers, the more likely his or her moral obligation would be translated into waste management behavior.

The present study also had some limitations. First, as this study used university students from China as a case, the findings may not be generalized to general populations (e.g., the residents) or populations in individualistic cultures. University students represent a specific young adult population with similar age and experiences in environmental learning. Hence, they may gain a better understanding of the environmental system and hold stronger environmental attitudes (Dunlap et al., 2000; Schwartz, 2009) in comparison with general populations. This may in turn, lead to different contours concerning the relationships of environmental knowledge and attitudes with waste management behavior between the two populations. Scholars also found that attitudes appeared to be more important in determining pro-environmental behaviors in individualistic cultures than it did in collectivistic cultures (Morren and Grinstein, 2016). Therefore, future research is recommended to examine the expanded TPB model in predicting waste management behavior from a comparative perspective, for example, comparing populations from different cultural settings, or comparing university students with residents within the same cultural setting. Second, because of the cross-sectional research design, this study could not examine the causal relationships between the variables in the model. It is recommended that future research use longitudinal or mixed research design to better identify how the changes in the variables would lead to waste management behavior. Third, noted that environmental knowledge was measured in an indirect way, the validity of the results in the present study might be influenced by the response bias. Future studies are encouraged to develop direct measures of knowledge in their investigations. It is also recommended that future studies involve both direct and indirect measures of knowledge to evaluate how different kinds of measures may influence the relationships between environmental knowledge and waste management behavior. Last but not the least, this study examined students’ waste management behavior through self-reported surveys. People are often biased when reporting on their own experiences as they are either consciously or unconsciously influenced by social desirability. Although some strategies (such as the anonymity and voluntariness of the online survey) were used to reduce the influence of social desirability, the social desirability bias could not be eradicated. In this regard, future studies on waste management behaviors are suggested to measure participants’ actual behavior using observation. For instance, Geng et al. (2015) employed a situational simulation experiment to measure participants’ spontaneous pro-environmental behavior by observing if they would use plastic bags to pack gifts when they completed the questionnaire. If the participant did not choose to use plastic bags, it was considered as environmentally friendly behavior. We suggest that future research use similar techniques as a supplementary instrument to support the interpretation of self-reported measures.

## Conclusion and Implications

The present study responded to a call for increased focus on waste management behavior of young adults by examining an expanded model of the TPB among university student populations in China. The findings suggest that the expanded TPB, with personal norms as the moral basis and environmental knowledge as the cognitive basis, would be a promising framework to understand waste management behavior of university students in China.

Drawing on the findings of this study, several practical implications are proposed. First, since subjective norms appear to be the most influential factor in determining waste management behavior of university student population, campus or social campaigns that target at university students should take the influence of their social networks (such as cohorts, friends, or interest groups) into consideration. This means that waste management initiatives should be promoted not only at the individual level, but also at the collective level through these social networks (especially the intimate network composed of important others) (White and Simpson, 2013). For example, campus recycling programs can encourage students or recruit youth leaders to share their recycling stories or tips or sustainable lifestyle through social networking or media platforms (e.g., WeChat, Twitter, TikTok, or Instagram). Such events can also be promoted offline in the form of workshops so that normative information can be disseminated among cohorts. In addition, programs with the purpose of inspiring information/knowledge sharing via social networks are suggested to be promoted from early stages of life when students start their school life at the primary level. Second, interventions should target students’ behavioral control over waste management practice so that they can gain confidence in their ability to overcome external barriers, and hence take actions. For instance, recycling propaganda or educational programs can use virtual reality technology to mimic local recycling scenarios in which students can gain recycling skills by “doing.” Since the higher level of environmental knowledge one has, the more likely he or she will perceive control over waste management behavior, information campaigns could be promoted to provide tips of performing waste management on campus in daily life or information concerning local waste management programs. Third, noted that environmental knowledge also offers an essential cognitive basis for developing moral motives for waste management behavior, educational program should highlight both environmental- and action-oriented knowledge to help university students gain thorough understanding of waste management from a critical and systematic perspective. For example, educational initiatives on recycling should not only provide information concerning procedures of recycling, but also help students gain system knowledge such as lifecycle of products from raw materials to waste treatment as well as environmental and social impacts involved in this process. Environmental courses can encourage students to participate in community recycling projects by using service-learning approaches so that they can gain a deeper understanding of interactions between ecological and sociopolitical systems (Hollweg et al., 2011).

## Data Availability Statement

The raw data supporting the conclusions of this article will be made available by the authors, without undue reservation.

## Ethics Statement

The studies involving human participants were reviewed and approved by Research Ethics Committee of Nantong University. Written informed consent for participation was not required for this study in accordance with the national legislation and the institutional requirements.

## Author Contributions

LW: research design, data collection and analysis, writing–theoretical framework, materials and methods, results, and discussion of original draft. YZ: questionnaire design and writing–introduction and discussion of original draft. JZ: writing–conclusion and implications of original draft, review, and editing. All authors contributed to the article and approved the submitted version.

## Conflict of Interest

The authors declare that the research was conducted in the absence of any commercial or financial relationships that could be construed as a potential conflict of interest.

## Publisher’s Note

All claims expressed in this article are solely those of the authors and do not necessarily represent those of their affiliated organizations, or those of the publisher, the editors and the reviewers. Any product that may be evaluated in this article, or claim that may be made by its manufacturer, is not guaranteed or endorsed by the publisher.

## Footnotes

1
www.wjx.cn
